# The influence of field size on stopping‐power ratios in‐ and out‐of‐field: quantitative data for the BrainLAB m3 micro‐multileaf collimator

**DOI:** 10.1120/jacmp.v13i6.4019

**Published:** 2012-11-08

**Authors:** M.L. Taylor, T. Kairn, T. Kron, L. Dunn, P.N. Johnston, R.D. Franich

**Affiliations:** ^1^ School of Applied Sciences and Health Innovations Research Institute RMIT University Melbourne Australia; ^2^ Physical Sciences Peter MacCallum Cancer Centre East Melbourne Australia; ^3^ William Buckland Radiotherapy Centre Alfred Hospital Melbourne Australia; ^4^ Premion Brisbane Australia; ^5^ Environmental and Radiation Health Branch Australian Radiation Protection and Nuclear Safety Agency Yallambie Australia

**Keywords:** stopping‐power ratio, stereotactic, small field, out‐of‐field, peripheral

## Abstract

The objective of this work is to quantify the systematic errors introduced by the common assumption of invariant secondary electron spectra with changing field sizes, as relevant to stereotactic radiotherapy and other treatment modes incorporating small beam segments delivered with a linac‐based stereotactic unit. The EGSnrc/BEAMnrc Monte Carlo radiation transport code was used to construct a dosimetrically‐matched model of a Varian 600C linear accelerator with mounted BrainLAB micro‐multileaf collimator. Stopping‐power ratios were calculated for field sizes ranging from $6\times 6\;{\text{mm}}^{2}$ up to the maximum ($98\times 98\;{\text{mm}}^{2}$), and differences between these and the reference field were computed. Quantitative stopping power data for the BrainLAB micro‐multileaf collimator has been compiled. Field size dependent differences to reference conditions increase with decreasing field size and increasing depth, but remain a fraction of a percent for all field sizes studied. However, for dosimetry outside the primary field, errors induced by the assumption of invariant electron spectra can be greater than 1%, increasing with field size. It is also shown that simplification of the Spencer‐Attix formulation by ignoring secondary electrons below the cutoff kinetic energy applied to the integration results in underestimation of stopping‐power ratios of about 0.3% (and is independent of field size and depth). This work is the first to quantify stopping powers from a BrainLAB micro‐multileaf collimator. Many earlier studies model simplified beams, ignoring collimator scatter, which is shown to significantly influence the spectrum. Importantly, we have confirmed that the assumption of unchanging electron spectra with varying field sizes is justifiable when performing (typical) in‐field dosimetry of stereotactic fields. Clinicians and physicists undertaking precise out‐of‐field measurements for the purposes of risk estimation, ought to be aware that the more pronounced spectral variation results in stopping powers (and hence doses) that differ more than for in‐field dosimetry.

PACS number: 87.10.RT; 87.53.Ly; 87.56.jf; 87.56.jk

## I. INTRODUCTION

Stopping‐power ratios are used in dosimetry protocols with a broad‐beam reference field and are assumed to be invariant with field size dependent changes in spectra. Earlier studies have shown field size effects to be small, though many of these do not explicitly include collimator scatter, which significantly influences spectra (the present work indicates a five‐fold increase of photons <500 keV due to collimator scatter). This work investigates the variation of stopping‐power ratios due to spectral changes in the context of small‐field radiotherapy using a linear accelerator with a micro‐multileaf collimator. This is done not only within the nominal treatment beam, but also out‐of‐field, which has received relatively little attention despite being of increasing contemporary interest.

Stereotactic radiosurgery involves small fields and single‐fraction high doses, and stereotactic radiotherapy often employs high doses (10–20 Gy per fraction) in a hypofractionated regime of few fractions. For such treatments, the necessity for accurate dosimetry is self‐evident, given the potential for detriment if the target is underdosed or if adjacent critical structures are subjected to excessive dose. However, the uncertainty of clinical dosimetry associated with stereotactic techniques and other methods (such as intensity‐modulated radiotherapy) that employ small beam segments, is greater than with conventional radiotherapy.^1^, ^3^ A detailed discussion has recently been provided by Alfonso et al.^(4)^ The importance and complexities of small‐field dosimetry are highlighted by the fact that much work is currently going into the development of reference dosimetry protocols specifically for small fields.^(5)^

The focus of the present study is that the stopping‐power ratios in dosimetry protocols acquired under broad‐beam conditions may be of questionable applicability in small‐field treatments, such as stereotactic radiotherapy. In this work, the effect of field size dependent spectral changes is analyzed in terms of its influence on stopping‐power ratios and, thus, ionization chamber calibration. Typically, a reference field of $10\times 10{\;\text{cm}}^{2}$ is used, and it is assumed that the difference in secondary electron spectrum under different conditions is negligible. However, although it is anticipated to be small, there is nonetheless a field size dependent change in spectrum that will influence absorbed dose calculations undertaken with an ionization chamber. It is the objective of the present study to quantify the magnitude of the systematic error that is induced by this assumption, in the context of linac‐based stereotactic fields.

Several authors have investigated aspects of this problem. Andreo and Brahme^(6)^ calculated stopping‐power ratios for several field sizes by determining secondary electron spectra resulting from Jessen's^(7)^ photon spectrum, obtained *via* thin‐film Compton‐scatter spectrometry. Circular photon fields were modeled and thus the field‐size dependence of spectra in this case is due to phantom scatter only, since collimation devices were not explicitly simulated. Andreo and Brahme found errors of up to 1% may be introduced by the assumption of field size independence. Heydarian et al.^(8)^ performed calculations of stopping‐power ratios as a function of depth, based on electron spectra obtained with EGS4, again using a precalculated broad‐beam photon data as input (i.e. collimation devices not modeled). Their method used mean electron energies to determine collision stopping‐power ratios, rather than the actual electron spectrum. Unlike the aforementioned studies, Verhaegen et al.^(2)^ performed explicit simulation (with EGS4) of a linear accelerator to obtain spectral data. They modeled a Varian 600SR with conical collimators (rather than X‐Y jaws); the tertiary collimator, a steel cone, varied in diameter from 1.25 to 5 cm. The Verhaegen study found little variation in secondary electron energies over this range. More recently, Sanchez‐Doblado et al.^(9)^ modeled an Elekta SL‐18 and Siemens Mevatron Primus; the former having conical collimators and the latter having a multileaf collimator (MLC) of 1 cm leaf width. They determined stopping‐power ratios for three different field sizes. Errors ranging between 0.3% and 1.1% were observed.

In this work, we undertake a highly systematic investigation of the dependence on field size and depth in the case of stereotactic fields shaped with a micro‐multileaf collimator on a Varian linac — a common combination.

There have been numerous studies concerned with the commissioning and implementation of stereotactic radiosurgery beams with micro‐multileaf collimators.^10^, ^12^ Belec et al.^(13)^ and Ding et al.^(14)^ have investigated the characteristics of stereotactic fields shaped with the BrainLAB m3 micro‐multileaf collimator (MMLC); however, a comprehensive investigation of spectral properties with varying field sizes and, in particular, the influence on stopping powers has not been undertaken. In our work, Monte Carlo methods have been employed to determine the spectral characteristics of a dosimetrically‐matched model of a Varian 600C with mounted BrainLAB MMLC. Calculated electron spectra in water are then used to evaluate stopping‐power ratios and thus determine the extent of the influence of changing spectra on absorbed dose calculations. Such investigations are not only undertaken within the primary field, but also out‐of‐field, which is of increasing interest in the context of out‐of‐field dose in stereotactic radiotherapy and associated risks of radiocarcinogenesis,^15^, ^16^ in particular for pediatric patients.^(17)^ Note that, in the present work, the term ‘primary field’ refers to the nominal treatment beam and 'out‐of‐field' refers to regions beyond the primary field.

## II. MATERIALS AND METHODS

### A. Stopping‐power ratio

It is possible to relate the dose to the gas in an ionization chamber to that in the medium of interest occupied by the dosimeter, such that the dose in the medium is given by:

(1) $$D_{med}=MN_{gas}{\left(\frac{\bar{L}}{\rho }\right)}_{gas}^{med}P_{ion}P_{rep1}P_{wall}$$

where *M* is the electrometer reading, $N_{\text{gas}}$ is the gas cavity calibration factor, and ${\left(\bar{L}/\rho \right)}_{\text{gas}}^{\text{med}}$ is the ratio of the mean restricted stopping power of the medium (phantom material) to that of the chamber gas (air). $P_{\text{ion}}$ is a factor that accounts for ionization recombination losses (the inverse of the ionization collection efficiency). $P_{\text{repl}}$ is a replacement correction depending on the type and energy of radiation, the gradient of the depth‐dose curve where the measurement is made and the radius of the chamber cavity. $P_{\text{wall}}$ is unity when the chamber wall and medium are of the same composition, and otherwise is a stopping power‐based correction which may be found elsewhere.^(18)^ These various factors account for the fact that the ionization chamber perturbs the dose field.

The ratio of the mean restricted stopping powers, may be given by:^(19)^

(2) $${\left(\frac{\bar{L}}{\rho }\right)}_{\text{gas}}^{\text{med}}=\frac{\int_{\Delta }^{E_{\max}}\Phi (E){\left(\frac{L_{\Delta }(E)}{\rho }\right)}_{med}\cdot dE+TE_{med}}{\int_{\Delta }^{E_{\max}}\Phi (E){\left(\frac{L_{\Delta }(E)}{\rho }\right)}_{gas}\cdot dE+TE_{gas}}$$

where, in this case, we will assume the medium (*med*) refers to water and the gas is air, Φ(*E*) is the energy spectrum of electrons, Δ is the cutoff energy, and *TE* is the track‐end term taking into account charged particles falling below Δ, given by: $\Phi (\Delta )\left[\frac{S_{\text{coll}}(\Delta )}{\rho }\right]\Delta$. It is instructive to read Nahum's(^20^) work which reinterprets the Spencer‐Attix(^19^) approach. The stopping‐power ratio is often assumed to be constant, since the variation in the energy spectrum in the case of broad‐beam photon irradiation is typically slight (for instance, calibration is performed with a $10\times 10{\;\text{cm}}^{2}$ field).

It is the objective of this work to quantify the dependence of field size and phantom depth on the stopping‐power ratio, so as to demonstrate the validity of the assumption of spectral invariance in small‐field applications, such as stereotactic radiotherapy. Monte Carlo methods are employed to explicitly investigate the extent of the influence of changing spectra on the calculated absorbed dose, and thus to identify any corresponding systematic errors that may be introduced.

### B. Monte Carlo calculations

A wide variety of Monte Carlo radiation transport codes are now available. One such code, EGSnrc,^(21)^ is interfaced with BEAMnrc,^(22)^ readily facilitating modeling of, in particular, radiotherapy linear accelerators. In this work we have constructed a model of the Varian 600C Clinac (Varian Medical Systems, Palo Alto, CA) with a mounted BrainLAB m3 micro‐multileaf collimator (MMLC) (BrainLAB, Feldkirchen, Germany) using BEAMnrc. The model was developed based on schematics provided by Varian Medical Systems and BrainLAB under nondisclosure agreements.^23^, ^24^ In this work, the model of the Varian 600C with MMLC was dosimetrically matched to measured data using percent depth‐dose curves, profiles, and scatter factors. A step size of 0.25 (maximum fractional energy loss, ESTEPE) was employed. EGSnrc has been shown to produce step‐size independent results at a sub 0.1% level even at interfaces of high Z media in fine geometries.^21^, ^25^ Here we have employed the PRESTA‐II electron‐step algorithm with the EXACT boundary crossing algorithm such that the electron transport will go into single‐scattering mode within three elastic mean free paths of the boundary, giving the necessary accuracy at peak efficiency. Calculations were performed on the VPAC Tango AMD Opteron system (VPAC Ltd., Carlton South VIC, Australia), which consists of 95 nodes, each with two AMD Barcelona 2.3 GHz quad core processors (totaling 760 cores). Typically, 15 processors were employed per simulation, each simulation thus requiring approximately 24 hours for 10^10^ incident particle histories.

In this work, simulations were undertaken for a range of depths in water and a systematic set of field sizes between 6times6 ${\text{mm}}^{2}$ and $98\times 98\;{\text{mm}}^{2}$ shaped with the micro‐multileaf collimator (with static open jaws). Stopping‐power ratios were calculated for points up to 20 cm off‐axis and up to 25 cm depth in water (relative to air). A commonly chosen value of 10 keV is taken for Δ, the influence of which is small (hence the value is often somewhat arbitrary). The track‐end term in Eq. (2) accounts for the energy deposition by particles falling below Δ. The statistical uncertainties in the calculated stopping‐power ratios range between 0.01% and 0.02%. It is important to note that these calculations were performed by explicitly following charged particles through the two media of interest, and not by scoring electron spectra and performing subsequent evaluation of the integral (Eq. (2)).

## III. RESULTS & DISCUSSION

The calculated values of ${\left(\bar{L}/\rho \right)}_{\text{gas}}^{\text{med}}$ are given in Table 1. The ratios for the various cases were compared against the calibration reference condition of a $9.8\times 9.8\;{\text{cm}}^{2}$ field — the largest possible (with the MMLC) and closest to $10\times 10{\;\text{cm}}^{2}$, as recommended by various protocols,^18^, ^26^, ^27^ at a depth (*d*) of 10 cm. This is represented by ${\left(\bar{L}/\rho \right)}_{R}$ in Eq. (3) below.

(3) $${\left(\frac{\bar{L}}{\rho }\right)}_{R}={\left[{\left(\frac{\bar{L}}{\rho }\right)}_{gas}^{med}\right]}_{FS,d}{\left[{\left(\frac{\bar{L}}{\rho }\right)}_{gas}^{med}\right]}_{FS=9.8\times 9.8,d=10}^{-1}$$

**Table 1:** The mean collisional stopping‐power ratio (where the medium med is water and the gas is air) for various field sizes (all at central axis) at various depths (from 5 to 25 cm). Statistical uncertainties are 0.01%‐0.02%.

*Field Size*	${\left(\bar{L}/\rho \right)}_{\text{gas}}^{\text{med}}$ *at Various Depths*				
	*5 cm Depth*	*10 cm Depth*	*15 cm Depth*	*20 cm Depth*	*25 cm Depth*
$6\times 6\;{\text{mm}}^{2}$	1.1195	1.1187	1.1176	1.1169	1.1161
$12\times 12\;{\text{mm}}^{2}$	1.1201	1.1194	1.1185	1.1176	1.1169
$18\times 18\;{\text{mm}}^{2}$	1.1205	1.1196	1.1189	1.1184	1.1176
$24\times 24\;{\text{mm}}^{2}$	1.1208	1.1201	1.1195	1.1188	1.1181
$30\times 30\;{\text{mm}}^{2}$	1.1210	1.1204	1.1195	1.1190	1.1184
$36\times 36\;{\text{mm}}^{2}$	1.1211	1.1205	1.1198	1.1192	1.1186
$42\times 42\;{\text{mm}}^{2}$	1.1213	1.1208	1.1202	1.1196	1.1190
$60\times 6.0\;{\text{mm}}^{2}$	1.1216	1.1212	1.1207	1.1198	1.1194
$80\times 80\;{\text{mm}}^{2}$	1.1217	1.1216	1.1213	1.1206	1.1199
$98\times 98\;{\text{mm}}^{2}$	1.1221	1.1218	1.1216	1.1211	1.1204

**Figure 1:**
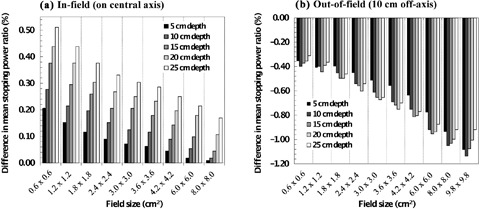
The difference in mean stopping power ratios, ${\left[{\left(\overset{―}{L}/\rho \right)}_{\text{gas}}^{\text{med}}\right]}_{diff}$ (defined in the main body text), between various field sizes and the reference case ($9.8\times 9.8\;{\text{cm}}^{2}$ at 10 cm depth), at (a) the central axis and (b) an out‐of‐field point 10 cm beyond the central axis. For the in‐field case, the discrepancy is clearly larger for smaller field sizes and greater depths, but is nonetheless less than 1%. For the out‐of‐field case, the discrepancy is larger for larger fields and exceeds 1% (take note that the differences are ‘opposite’, hence negative).

This is given in Table 2 for the various field sizes (FS). To illustrate the extent of the discrepancy for different field sizes and depths, the percentage difference is plotted in Fig. 1, where the difference (for various field sizes, FS, and depths, (*d*) is defined as:

(4) $${\left[{\left(\bar{L}/\rho \right)}_{gas}^{med}\right]}_{diff}(\%)=100\left({\left[{\left(\bar{L}/\rho \right)}_{gas}^{med}\right]}_{FS=9.8\times 9.8,d=10}-{\left[{\left(\bar{L}/\rho \right)}_{gas}^{med}\right]}_{FS,d}\right){\left({\left[{\left(\bar{L}/\rho \right)}_{gas}^{med}\right]}_{FS,d}\right)}^{-1}$$

**Table 2:** The ratio of mean collisional stopping‐power ratios (where the medium med is water and the gas is air) for various field sizes (all at central axis) at various depths (from 5 to 25 cm), relative to the reference case of field size $9.8\times 9.8{\text{cm}}^{2}$ and depth 10 cm. Statistical uncertainties are 0.01%–0.03%.

*Field Size*	${\left(\bar{L}/\rho \right)}_{R}$ *at Various Depths*				
	*5 cm Depth*	*10 cm Depth*	*15 cm Depth*	*20 cm Depth*	*25 cm Depth*
$6\times 6\;{\text{mm}}^{2}$	0.9979	0.9972	0.9963	0.9956	0.9949
$12\times 12\;{\text{mm}}^{2}$	0.9985	0.9979	0.9971	0.9963	0.9956
$18\times 18\;{\text{mm}}^{2}$	0.9988	0.9980	0.9974	0.9970	0.9963
$24\times 24\;{\text{mm}}^{2}$	0.9991	0.9985	0.9979	0.9973	0.9967
$30\times 30\;{\text{mm}}^{2}$	0.9993	0.9988	0.9979	0.9975	0.9970
$36\times 36\;{\text{mm}}^{2}$	0.9994	0.9988	0.9982	0.9977	0.9971
$42\times 42\;{\text{mm}}^{2}$	0.9996	0.9991	0.9986	0.9980	0.9975
$60\times 6.0\;{\text{mm}}^{2}$	0.9998	0.9995	0.9990	0.9982	0.9979
$80\times 80\;{\text{mm}}^{2}$	0.9999	0.9998	0.9996	0.9989	0.9983
$98\times 98\;{\text{mm}}^{2}$	1.0003	1.0000	0.9998	0.9994	0.9988

**Figure 2:**
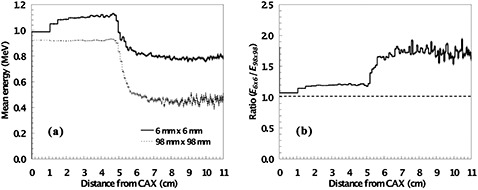
The mean energy (a) as a function of distance from the central axis (CAX) for a $6\times 6\;{\text{mm}}^{2}$ field (solid line) and a $98\times 98\;{\text{mm}}^{2}$ field (broken line). The ratio of the mean energy (b) of the small field to the large field as a function of distance from the central axis. The jaws are set to a static $98\times 98\;{\text{mm}}^{2}$ opening. The purpose of this to demonstrate that although the mean energy of the secondary electron spectrum within the primary field will be similar for (significantly) different field sizes, beyond the primary beam, discrepancies of almost a factor of two may exist.

The results in (Fig. 1a) clearly show that although there is a spectral change, it is not significant enough to generate discrepancies greater than roughly half a percent relative to the reference condition (for the range of field sizes and depths studied here). It should be noted that the results in (Fig. 1a) correspond only to the primary field, and differences may be more pronounced for out‐of‐field measurements. This point is highlighted by Fig. 2.

In the case of out‐of‐field spectra, there are notable differences compared to the in‐field case which significantly influence the stopping powers calculated. An example of this difference in electron spectra in‐ and out‐of‐field is illustrated by Fig. 2, which shows the mean energy (evaluated from energy fluence) as a function of distance from the central axis, as well as the ratio of mean energy for the 6times6 ${\text{mm}}^{2}$ relative to the $98\times 98\;{\text{mm}}^{2}$ field. The discrepancy in mean energy is clearly larger in the region beyond about 5–6 cm off‐axis. An equivalent set

of ${\left(\bar{L}/\rho \right)}_{\text{gas}}^{\text{med}}$ calculations at a representative point 10 cm off‐axis were undertaken. To compare these to ${\left(\bar{L}/\rho \right)}_{\text{gas}}^{\text{med}}$ for the $98\times 98\;{\text{mm}}^{2}$ primary field, percentage differences, ${\left[{\left(\bar{L}/\rho \right)}_{\text{gas}}^{\text{med}}\right]}_{\text{dif}f}$ were evaluated. These results are shown in (Fig. 1b) and summarized in Table 3. Confidence in the results obtained are strengthened by the fact that the trend agrees qualitatively with that published by Eklund and Ahnesjö.^(28)^

**Table 3:** The mean restricted collisional stopping‐power ratios (med refers to water and gas is air) for the out‐of‐field spectra (10 cm off‐axis) various field sizes. Also shown is the percentage difference compared to the reference 98 X 98 mm^2^ field at central axis. These are shown for depths from 5–20 cm in water. Note in particular that the percentage differences are in this case negative, unlike the difference for a primary field comparison. (Please refer to the main body text for definition of the parameters in this table.)

*Field Size*	$\left[{\left(\bar{L}/\rho \right)}_{\text{gas}}^{\text{med}}\right]$ *at Various Depths*				${\left[{\left(\bar{L}/\rho \right)}_{\text{gas}}^{\text{med}}\right]}_{diff}$ *(%) at Various Depths*			
	*5 cm*	*10 cm*	*15 cm*	*20 cm*	*5 cm*	*10 cm*	*15 cm*	*20 cm*
$6\times 6\;{\text{mm}}^{2}$	1.1258	1.1263	1.1260	1.1258	‐0.355	‐0.400	‐0.373	‐0.355
$12\times 12\;{\text{mm}}^{2}$	1.1264	1.1263	1.1268	1.1262	‐0.408	‐0.400	‐0.444	‐0.391
$18\times 18\;{\text{mm}}^{2}$	1.1263	1.1269	1.1274	1.1274	‐0.400	‐0.453	‐0.497	‐0.497
$24\times 24\;{\text{mm}}^{2}$	1.1269	1.1279	1.1281	1.1286	‐0.453	‐0.541	‐0.558	‐0.603
$30\times 30\;{\text{mm}}^{2}$	1.1276	1.1287	1.1292	1.1294	‐0.514	‐0.611	‐0.655	‐0.673
$36\times 36\;{\text{mm}}^{2}$	1.1281	1.1296	1.1299	1.1303	‐0.558	‐0.691	‐0.717	‐0.752
$42\times 42\;{\text{mm}}^{2}$	1.129	1.1303	1.1310	1.1309	‐0.638	‐0.752	‐0.813	‐0.805
$60\times 6.0\;{\text{mm}}^{2}$	1.1306	1.1322	1.1326	1.1324	‐0.778	‐0.919	‐0.954	‐0.936
$80\times 80\;{\text{mm}}^{2}$	1.1324	1.1337	1.1335	1.1331	‐0.936	‐1.050	‐1.032	‐0.997
$98\times 98\;{\text{mm}}^{2}$	1.1341	1.1347	1.1340	1.1332	‐1.085	‐1.137	‐1.076	‐1.006

Although the difference between the two is opposite (i.e., negative) to the primary field comparison observed earlier, the difference is still nonetheless <1% for the case of small fields. However, this is the case purely because although the small‐field out‐of‐field electron spectrum differs from the primary (in‐field) beam spectrum, it happens to be close to that of the large‐field primary beam spectrum. For the larger fields, the differences are more pronounced.

Referring to (Fig. 2a), it is clear that the mean out‐of‐field energy for the small‐field case is comparable to the mean energy of the primary beam of the $98\times 98\;{\text{mm}}^{2}$ field. Unlike the previous cases, where small fields have exhibited the largest discrepancy, (Fig. 2a) indicates that for out‐of‐field measurements, large fields might be more problematic (the mean out‐of‐field energy for the large field is approximately half that of the primary field). As such, stopping‐power ratios have also been calculated for the out‐of‐field electron spectra for the range of fields (see (Fig. 1b). Data are also summarized in Table 3. The discrepancy is much more pronounced for out‐of‐field spectra for larger fields — and may exceed 1%. The effect of the static jaw size of $98\times 98\;{\text{mm}}^{2}$ is clear in Fig. 2. It is worth noting that using backed‐up jaws (data not shown), compared to that shown in Fig. 2, results in a relatively lower mean energy in the region shadowed by the MMLC, but a relatively higher mean energy beyond the off‐axis distance of ~ 5 cm.

For the vast majority of applications, the approximation of a constant stopping‐power ratio obtained with use of a broad beam (typically $10\times 10{\;\text{cm}}^{2}$) is generally acceptable, since the associated error is small (a fraction of a percent). It should be noted, however, that in the context of out‐of‐field dosimetry, the same assumption results in discrepancies <1%. For dedicated stereotactic units, the use of a smaller reference field (e.g., $5\times 5{\;\text{cm}}^{2}$) is feasible and could be considered as a means of reducing this error. The contributions to dosimetric uncertainties have been quantified and presented; each contribution to the discrepancy between planned and delivered doses should be known as well as possible. The clinical impact of dosimetric uncertainty is likely to be site/anatomy/patient‐specific and, in the context of radiation protection and risk estimation, dependent on the patient treatment load.

It is also of interest to consider the influence of the track‐end term in Eq. (2). Stopping‐power ratios were also calculated without incorporating charged particles below the energy threshold Δ; let this be referred to as ${\left[{\left(\overset{―}{L}/\rho \right)}_{\text{gas}}^{\text{med}}\right]}_{\text{simplified}}$ for clarity. This was undertaken for the same set of field sizes (ranging from 6times6 ${\text{mm}}^{2}$ to $98\;\times \;98\;{\text{mm}}^{2}$) for depths of 5, 10, and 15 cm. The data obtained were compared to that obtained via Eq. (2) in terms of percentage difference:

(5) $${\left[{\left(\frac{\bar{L}}{\rho }\right)}_{gas}^{med}\right]}_{comparison}(\%)=100\left({\left[{\left(\frac{\bar{L}}{\rho }\right)}_{gas}^{med}\right]}_{simplified}-\left[{\left(\frac{\bar{L}}{\rho }\right)}_{gas}^{med}\right]\right){\left([{\left(\frac{\bar{L}}{\rho }\right)}_{gas}^{med}]\right)}^{-1}$$

**Figure 3:**
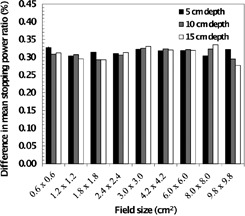
The difference, ${\left[{\left(\left(\overset{―}{L}/\rho \right)\right)}_{\text{gas}}^{\text{med}}\right]}_{comparison}$, between stopping‐power ratios calculated via the Spencer‐Attix approach (Eq. (2)) and a simplified approach. In the latter case, the influence of particles of kinetic energy below the cut‐off of Δ=10 keV is ignored. There is only slight field size and depth dependence.

The values calculated with Eq. (5) are plotted in Fig. 3. It is clear from the latter figure that the simplified approach consistently underestimates the stopping‐power ratios compared to the Spencer‐Attix approach, exhibiting a mean difference of 0.313 (σ=0.014 )%, with little dependence on field size and depth.

## IV. CONCLUSIONS

Stopping‐power ratios are used in dosimetry protocols with a broad‐beam reference field and are assumed to be invariant with field size dependent changes in spectra. A number of studies have demonstrated that field size effects are small; however, many of these did not explicitly include collimator scatter, which has a significant influence on spectra (greatly increased low‐energy photon fluence). In this work, we have constructed a dosimetrically matched Monte Carlo radiation transport model of a linear accelerator with micro‐multileaf collimator and used this to compute stopping‐power ratios for a number of field sizes (between 6times6 ${\text{mm}}^{2}$ and $98\;\times \;98\;{\text{mm}}^{2}$) and depths (between 5 and 25 cm). Errors arising due to the assumption of field size independent stopping‐power ratios in the context of small fields relevant to stereotactic radiotherapy have been shown to be small. Discrepancies relative to the reference field case increase with decreasing field size but, nonetheless, remain within a fraction of a percent. However, we also evaluated stopping‐power ratios outside the nominal field (i.e., out‐of‐field) and found that, for larger field sizes in particular, there may be a systematic error <1% attributable to the assumption of unchanging electron spectra for different field sizes and spatial locations within the phantom. For typical clinical work, this is not likely to be of great concern (since the dose under the jaws is ~ 0.1%). However, it is of interest when assessing doses outside the primary field in stereotactic radiotherapy for applications such as risk estimation.^15^, ^17^ This will affect absorbed dose calculations derived using such stopping‐power ratios, and may be less readily accounted for than (field size independent) issues such as leakage that are typically encountered in out‐of‐field dosimetry. Dosimeters with large energy dependence may be more significantly affected. It is also noted that ignoring the influence of secondary electrons generated below the A energy cut‐off results in slight underestimation of stopping‐power ratios (by about ~ 0.3%), and does not exhibit systematic dependencies on either field size or depth.

## ACKNOWLEDGMENTS

This work is supported by National Health and Medical Research Council (NHMRC, Australia) Project Grant 555240. The authors would like to thank Dr. J. Lydon and D. Butler for providing useful comments on the manuscript.

## References

[1] Das I , Ding G , Ahnesjo A . Small fields: nonequilibrium radiation dosimetry. Med Phys. 2008;35(1):206–15. 18293576 10.1118/1.2815356

[2] Verhaegen F , Das I , Palmans H . Monte Carlo dosimetry study of a 6 MV stereotactic unit. Phys Med Biol. 1998;43(10):2755–68. 9814515 10.1088/0031-9155/43/10/006

[3] Taylor ML , Kron T , Franich RD . A contemporary review of stereotactic radiotherapy: inherent dosimetric complexities and the potential for detriment. Acta Oncol. 2011;50(4):483–508. 21288161 10.3109/0284186X.2010.551665

[4] Alfonso R , Andreo P , Capote R , et al. A new formalism for reference dosimetry of small and nonstandard fields. Med Phys. 2008;35(11):5179–86. 19070252 10.1118/1.3005481

[5] Alfonso R , Andreo P , Capote R , et al. Present status of IAEA/AAPM recommendations on small and composite field dosimetry [abstract]. Med Phys. 2010;37:3096.

[6] Andreo P and Brahme A . Stopping power data for high‐energy photon beams. Phys Med Biol. 1986;31(8):839–58. 3094045 10.1088/0031-9155/31/8/002

[7] Jessen K . Measurements of primary spectra from a kilocurie 60Co unit and a 6 MeV linear accelerator. Acta Radiol Ther Phys Biol. 1973;12(6):561–68. 4207002 10.3109/02841867309130421

[8] Heydarian A , Hoban P , Beddoe A . A comparison of dosimetry techniques in stereotactic radiosurgery. Phys Med Biol. 1996;41(1):93–110. 8685261 10.1088/0031-9155/41/1/008

[9] Sanchez‐Doblado F , Andreo P , Capote R , et al. Ionization chamber dosimetry of small photon fields: a Monte Carlo study on stopping‐power ratios for radiosurgery and IMRT beams. Phys Med Biol. 2003;48(14):2081–99. 12894972 10.1088/0031-9155/48/14/304

[10] Benedict S , Carnidale R , Wu Q , Zwicker R , Broaddus W , Mohan R . Intensity‐modulated stereotactic radiosurgery using dynamic micro‐multileaf collimation. Int J Radiat Oncol Biol Phys. 2001;50(3):751–58. 11395244 10.1016/s0360-3016(01)01487-0

[11] Cosgrove V , Jahn U , Pfaender M , Bauer S , Budach V , Wurm R . Commissioning of a micro multi‐leaf collimator and planning system for stereotactic radiosurgery. Radiother Oncol. 1999;50(3):325–36. 10392819 10.1016/s0167-8140(99)00020-1

[12] Deng J , Guerrero T , Ma C , Nath R . Modelling 6 MV photon beams of a stereotactic radiosurgery system for Monte Carlo treatment planning. Phys Med Biol. 2004;49(9):1689–704. 15152924 10.1088/0031-9155/49/9/007

[13] Belec J , Patrocinio H , Verhaegen F . Development of a Monte Carlo model for the Brainlab microMLC. Phys Med Biol. 2005;50(5):787–99. 15798255 10.1088/0031-9155/50/5/005

[14] Ding G , Duggan D , Coffey C . Commissioning stereotactic radiosurgery beams using both experimental and theoretical methods. Phys Med Biol. 2006;51(10):2549–66. 16675869 10.1088/0031-9155/51/10/013

[15] Taylor ML , McDermott LN , Johnston PN , et al. Stereotactic fields shaped with a micro‐multileaf collimator: systematic characterization of peripheral dose. Phys Med Biol. 2010;55(3):873–81. 20071767 10.1088/0031-9155/55/3/021

[16] Taylor ML and Kron T . Consideration of the radiation dose delivered away from the treatment field to patients in radiotherapy. J Med Phys. 2011;36(2):59–71. 21731221 10.4103/0971-6203.79686 PMC3119954

[17] Taylor ML , Kron T , Franich RD . Assessment of out‐of‐field doses in radiotherapy of brain lesions in children. Int J Radiat Oncol Biol Phys. 2011;79(3):927–33. 20732763 10.1016/j.ijrobp.2010.04.064

[18] A protocol for the determination of absorbed dose from high‐energy photon and electron beams. Med Phys. 1983;10(6):741–71. 6419029 10.1118/1.595446

[19] Spencer L and Attix F . A theory of cavity ionization. Radiat Res. 1955;3(3):239–54. 13273628

[20] Nahum A . Water/air mass stopping power ratios for megavoltage photon and electron beams. Phys Med Biol. 1978;23(1):24–38. 416446 10.1088/0031-9155/23/1/002

[21] Kawrakow I . Accurate condensed history Monte Carlo simulation of electron transport: I. EGSnrc, the new EGS4 version. Med Phys. 2000;27(3):485–98. 10757601 10.1118/1.598917

[22] Rogers D , Walter B , Kawrakow I . BEAMnrc users manual. PIRS‐0509. Ottawa, Canada: NRCC; 2007.

[23] Kairn T , Aland T , Franich R , et al. Adapting a generic BEAMnrc model of the BrainLAB m3 micro‐multileaf collimator to simulate a local collimation device. Phys Med Biol. 2010;55(17):N451–N463. 20702922 10.1088/0031-9155/55/17/N01

[24] Kairn T , Kenny J , Crowe SF , et al. Modeling a complex micro‐multileaf collimator using the standard BEAMnrc distribution. Med Phys. 2010;37(4):1761–67. 20443498 10.1118/1.3355873

[25] Verhaegen F . Evaluation of the EGSnrc Monte Carlo code for interface dosimetry near high‐Z media exposed to kilovolt and 60Co photons. Phys Med Biol. 2002;47(10):1691–705. 12069087 10.1088/0031-9155/47/10/306

[26] Almond P , Biggs P , Coursey B , et al. AAPM's TG‐51 protocol for clinical reference dosimetry of high‐energy photon and electron beams. Med Phys. 1999;26(9):1847–70. 10505874 10.1118/1.598691

[27] IAEA . Absorbed dose determination in external beam radiotherapy. TRS 398. Vienna: IAEA; 2000.

[28] Eklund K and Ahnesjo A . Fast modelling of spectra and stopping‐power ratios using differentiated fluence pencil kernels. Phys Med Biol. 2008;53(16):4231–47. 18653924 10.1088/0031-9155/53/16/002

